# Assessing the role of sodium fluorescein in peripheral nerve sheath tumors and mimicking lesions surgery: An update after 142 cases

**DOI:** 10.3389/fonc.2022.1070878

**Published:** 2023-01-09

**Authors:** Vittoria Nazzi, Niccolò Innocenti, Nicolò Castelli, Irene Tramacere, Marica Eoli, Vittoria Cojazzi, Laura Gatti, Francesco Acerbi, Jacopo Falco, Ignazio G. Vetrano

**Affiliations:** ^1^ Department of Neurosurgery, Fondazione IRCCS Istituto Neurologico Carlo Besta, Milan, Italy; ^2^ Department of Research and Clinical Development, Scientific Directorate, Fondazione IRCCS Istituto Neurologico Carlo Besta, Milan, Italy; ^3^ Molecular Neuro-Oncology Unit, Fondazione IRCCS Istituto Neurologico Carlo Besta, Milan, Italy; ^4^ Neurobiology Laboratory, Fondazione IRCCS Istituto Neurologico Carlo Besta, Milan, Italy; ^5^ Department of Biomedical Sciences for Health, Università degli Studi di Milano, Milan, Italy

**Keywords:** fluorescein-guided surgery, peripheral nerve sheath tumors, PNST, schwannoma, neurofibroma, sodium fluorescein (SF), YELLOW560 filter

## Abstract

**Objective:**

Peripheral nerve sheath tumors (PNST) include mainly schwannomas and neurofibromas. Surgical resection represents the mainstay of treatment but due to their pathogenesis, distinguishing between intact functional nerve and the fibers from whence the PNST arose may not always be easy to perform, constituting the most relevant risk factor in determining a worsening in neurological condition. The introduction of intraoperative tools to better visualize these tumors could help achieve a gross-total resection. In this study, we analyzed the effect of sodium fluorescein (SF) on the visualization and resection of a large cohort of PNST.

**Methods:**

Between September 2018 and December 2021, 142 consecutive patients harboring a suspected PNST underwent fluorescein-guided surgery at the Department of Neurosurgery of the Fondazione IRCCS Istituto Neurologico Carlo Besta, Milan, Italy. All patients presented with a different degree of contrast enhancement at preoperative MRI. SF was intravenously injected after intubation at 1 mg/kg. Intraoperative fluorescein characteristics and postoperative neurological and radiological outcomes were collected, analyzed, and retrospectively compared with a historical series.

**Results:**

142 patients were included (42 syndromic and 100 sporadic); schwannoma was the predominant histology, followed by neurofibroma (17 neurofibroma e 12 plexiform neurofibroma) and MPNST. Bright fluorescence was present in all cases of schwannomas and neurofibromas, although with a less homogeneous pattern, whereas it was significantly less evident for malignant PNST; perineurioma and hybrid nerve sheath tumors were characterized by a faint fluorescence enhancement. The surgical resection rate in the general population and even among the subgroups was about 66.7%; from the comparative analysis, we found a consistently higher rate of complete tumor removal in plexiform neurofibromas, 66% in the “fluorescent” group vs 44% in the “historical” group (p-value < 0.05). The rate of complications and mean surgical time were superimposable among the two populations.

**Conclusions:**

SF is a valuable method for safe fluorescence-guided PNST and mimicking lesions resection. Our data showed a positive effect of fluorescein-guided surgery in increasing the rate of surgical resection of plexiform neurofibromas, suggesting a possible role in improving the functional and oncological outcome of these lesions.

## 1 Introduction

Peripheral nerve sheath tumors (PNST) constitute a miscellaneous group that counts for 10-15% of all soft tissue primary tumors (1). Among the histopathologic entities within the PNST (2), benign lesions, i.e., schwannomas and neurofibromas, are the most common overall and more frequently affect adults (3, 4). PNST can be sporadic lesions or associated with neurocutaneous syndromes characterized by multisystemic manifestations, prominently affecting structures primarily derived from the ectoderm (5, 6). Syndromic PNST affect young adults and children and are generally present in multiple localizations, including several subcutaneous lesions; although it is a rare subgroup, malignant PNST are more frequent in syndromic diseases than sporadic conditions. Neurofibromas are mainly related to neurofibromatosis type 1 (NF1), and they present the risk of malignant transformation into aggressive and infiltrative sarcomas (7, 8), with severe morbidity and also mortality (9). The role of adjuvant radiotherapy and traditional chemotherapy remains unclear for such tumors. The mainstay of treatment is surgical enucleation which should not lead to neurological impairment (3, 10, 11); gross-total resection is a pivotal predictor of local control and recurrence and could prevent possible malignant behavior (12). A relevant tool to reduce surgical morbidity on neurological functions is the use of intraoperative neurological monitoring (IOM) (13) which has an impact on identifying functional nerves, localizing the safest entry point inside the tumor capsule, and recognizing anatomical structures in case of large and destructive tumors. Nevertheless, the role of IOM is still an argument of debate among different groups involved in PNST surgery (14). Moreover, distinguishing between functional intact nerves and the silent fibers from whence the PNST arose is not always easy to perform (10). As already studied in central nervous system (CNS) tumors surgery, using intraoperative fluorophores could represent a valuable method to visualize pathological tissue better and possibly improve tumor resection (15–18).

Fluorescein is a green fluorescent synthetic organic compound with countless medical applications, mainly as fluorescein sodium salt (SF), a water-soluble dye. Although some pioneering applications in neuro-oncology (19), its use has been initially limited to ophthalmology (20). However, introducing a dedicated and integrated filter in the surgical microscope has exponentially increased SF applications in neurosurgery. After the first prospective phase II trial to evaluate the safety and efficacy of fluorescein-guided resection of high-grade gliomas by the use of a dedicated fluorescence filter in the surgical microscope (17), the Italian Medicine Agency (AIFA) has extended the indications for the utilization of fluorescein molecule (905/2015) (21). According to this determination, the use of SF as a neurosurgical tracer during oncological procedures is approved, and the Italian National Health System reimburses its cost.

The potential role of SF in PNST surgery is still little explored, despite some preliminary clinical reports (22–25). Experimental models have demonstrated that SF causes endoneurial extravasation, like blood-brain barrier extravasation, confirming the behavior of fluorescein as an unspecific vascular dye (26–28). In 2019, we presented our preliminary experience in a series of 20 patients with different PNST, in which fluorescein was used for intraoperative visualization and guidance in tumor removal (25), and we demonstrated that fluorescein is a feasible, safe, and helpful intraoperative adjunct to better identify and distinguish PNST from intact functional nerves, with a possible impact on tumor resection, particularly in diffuse neurofibromas (29, 30). Then we started a prospective analysis on a broader series regarding the use of SF as an intraoperative tracer for peripheral nerve tumors. We present our experience and benefits of intraoperative SF application during surgical removal of different PNST.

## 2 Methods

### 2.1 Patients and inclusion criteria

The inclusion criteria were: (1) patients of both genders, excluding pediatric patients (2) patients with suspected PNST, as suggested by preoperative ultrasound and MRI with paramagnetic contrast agent administration. The exclusion criteria were: (1) impossibility to give consent due to cognitive deficits or language disorders; (2) known allergy to contrast agents or history of previous anaphylactic shocks or known severe previous adverse reactions to SF; (3) acute myocardial infarction or stroke in the last 90 days; (4) severe organ failure; (5) women in their first trimester of pregnancy or lactation. Patients harboring mimicking lesions at histopathological examination but who had required a similar microsurgical treatment including the role of intraoperative neuromonitoring to preserve neurological function have not been excluded. The study started in September 2018, when the first patient was enrolled. All patients gave written informed consent for the surgical procedure, including the use of fluorescein, and were included in the prospective study about the use of fluorescein in neurosurgical procedures. The protocol has been approved by the Ethical Committee of the Fondazione IRCCS Istituto Neurologico Carlo Besta. To identify a comparative cohort of patients affected by PNST undergoing surgery before SF diffusion, we systematically and retrospectively reviewed our departmental surgical and neuropathological databases between March 2015 and August 2018. The Institutional review board approved the retrospective analysis.

### 2.2 Clinical and radiological management

The preoperative assessment included physical and neurological examination, laboratory test results, preoperative contrast-enhanced MRI of the region of interest, and ultrasonography. The postoperative clinical evaluation comprises neurological examination as above, laboratory test (kidney function), and exclusion of any side effect related to fluorescein injection. Patients with sporadic PNST received standard radiological follow-up with a first MRI of the nerve and body segment of interest three months after surgery; on the contrary, patients affected by neurofibromatosis or schwannomatosis underwent a more accurate neuro-oncological follow-up, also comprising brain and spinal MRI. Gross total resection was defined as the absence of residual hyperintense tissue on postoperative postcontrast volumetric T1 images after subtraction of the volume of spontaneous hyperintense tissue in volumetric T1 images without contrast; the T2 sequence was further analyzed and compared to preoperative one to exclude residual pathological tissue.

### 2.3 Surgical protocol

The standardized surgical protocol of fluorescein-guided technique is based on intravenous (i.v.) SF (*Monico S.p.A., Venice, Italy*) injection at the standard dose of 1mg/kg immediately upon completion of the induction of general anesthesia. Surgery was performed under microscopic view through surgical microscopes equipped with an integrated fluorescent filter tailored to sodium fluorescein excitation and emission wavelength (YELLOW 560 – *Pentero 900; Carl Zeiss Meditec, Oberkochen, Germany*). During resection, the microscope could be switched alternatively from fluorescent to white-light illumination. The safest surgical technique forecasts a sufficiently large tumor exposure, followed by inspection and pseudocapsule or true capsule direct electrophysiological stimulation to identify possible functional fascicles running inside. To guarantee an accurate IOM, general anesthesia is administered in a totally intravenous way with a targeted controlled infusion (TIVA-TCI) of a combination of propofol and remifentanil, maintained at a constant plasma concentration due to pharmacokinetic models; muscle relaxants are only used during intubation and, when necessary, a curare antagonist is administered before starting the electrostimulation. The subsequent surgical step is, therefore, the incision of a safe, silent entry zone, followed by intraneural and longitudinal dissection allowing progressive isolation and tumor removal *via* en bloc enucleation. In the case of huge tumors or neurofibromas, neoplastic tissue is usually removed piecemeal when an en-bloc enucleation is not feasible. Current neurophysiological protocol for peripheral nerve surgery comprises continuous free-running and stimulus-triggered electromyography to verify during and at the end of the procedure the functional integrity of the nerve.

### 2.4 Intraoperative fluorescence characteristics and side effects

Fluorescence intensity was subjectively graded by the surgeon as bright (homogeneous or inhomogeneous), faint or absent during surgical procedure; in addition, it was reported by the surgeon whether fluorescein was considered useful for removing the tumor. Furthermore, medical reports were evaluated for any possible adverse effect or allergic reaction to fluorescein administration.

### 2.5 Histological analysis

Histopathological analysis was performed in each case; tumors were classified according to the 2016 and 2021 WHO classification of nervous tissue tumors (31, 32) by the neuro-pathology group of our Institute as part of the daily clinical practice.

### 2.6 Statistical analysis

The sample was described by means of the usual descriptive statistics: mean, median, and standard deviation for continuous variables and proportions for categorical ones. PRISM software for Macintosh was used for the statistical analysis.

## 3 Results

A total of 142 consecutive patients (75 females and 67 males) admitted at our Institution between September 2018 and December 2021 for a suspected PNST were included in the analysis. The mean age of presentation was 45.1 years, with a median age of 47.8 years (ranging from 18 to 82 years). The median follow-up in our cohort was 6.5 months, with a minimum of 3 months and a maximum of 35 months. Patients with neurocutaneous syndromes represented 29.6% of our cohort (42/142): 21 patients (14.8%) were affected by NF1, 15 patients (10.6%) by schwannomatosis, and 6 (4.2%) by NF2. The most common neoplasms were schwannomas (92/142 – 64.8%), followed by neurofibromas (17/142 – 12%) and plexiform neurofibromas (12/142 – 8.5%); MPNST were the fourth most represented histology (5/142 – 3.5%). The remaining neoplasms were a heterogeneous group with less common PNST, such as hybrid peripheral nerve tumors, perineurioma, lymphomas, and others. Histopathological and intraoperative fluorescein findings are reported in **Table 1**.

**Table 1:** Clinical characteristics of the Sodium Fluorescein series.

Histology	N°	Neurocutaneous syndromes	Location	Resection	Complications
**Schwannoma**	91 (64%)	9 Schwannomatosis 7 NF2 3 NF1	39 Inferior Limbs 21 Superior limbs 18 Intracanalar 12 Brachial Plexus	87/91 Total (95,6%) 4/91 Subtotal (4,4%)	4 transient sensory deficits 2 transient motor deficits 1 dural tear
**Neurofibroma**	17 (12%)	10 NF1	7 Intracanalar 4 Head 3 Superior limbs 2 Back 1 Inferior limbs	15/17 Total (88%) 2/17 Subtotal (12%)	1 transient sensory deficit 1 permanent motor deficit
**Plexiform Neurofibroma**	12 (8.5%)	8 NF1 3 Rasopathy	5 Head 5 Inferior limbs 1 Brachial plexus 1 Superior limbs	8/12 Total (66%) 4/12 Subtotal 34%)	1 worsening of a pre-existing hypostenia
**Malignant Peripheral Nerve Sheath Tumor (MPSNT)**	5 (3.5%)	1 NF2	4 Brachial Plexus 1 Intracanalar	1/5 Total (20%) 4/5 Subtotal (80%)	no
**Hybrid PNS tumor**	3	2 Schwannomatosis 1 Rasopathy	2 Superior limbs 1 Inferior limbs	1/3 Total (33%) 2/3 Subtotal (66%)	1 transient motor deficit
**Follicular lymphoma**	2	none	2 Superior limbs	Total	no
**LN hyperplasia**	1	none	Superior limbs	Total	no
**Plexiform Schwannoma**	1	none	Inferior limbs	Total	no
**Ganglioneuroma**	1	none	Intracanalar	Subtotal	no
**Lypomatosis of the nerve**	1	none	Superior limbs	Subtotal	no
**Perineurioma**	1	none	Intracanalar	Subtotal	no
**Steatonecrosis**	1	none	Inferior limbs	Subtotal	no
**Ganglion cyst**	1	none	Inferior limbs	Total	no
**Epithelioid hemangioendothelioma**	1	none	Superior limbs	Total	no
**Mesenchymal tissue tumor**	1	none	Brachial Plexus	Total	no
**Nodular tenosynovitis**	1	none	Inferior limbs	Total	no
**Solitary fibrous tumor**	1	none	Superior limbs	Total	no
**Mixopapillary Ependimoma**	1	none	Intracanalar	Total	no
**Total**	142	21 NF1 12 Schwannomatosis 8 NF2 4 Rasopathies	49 Inferior limbs 33 Superior limbs	122/142 Total (86%) 20/142 Subtotal (14%)	11/142 (7.7%)

**Figure 1:**
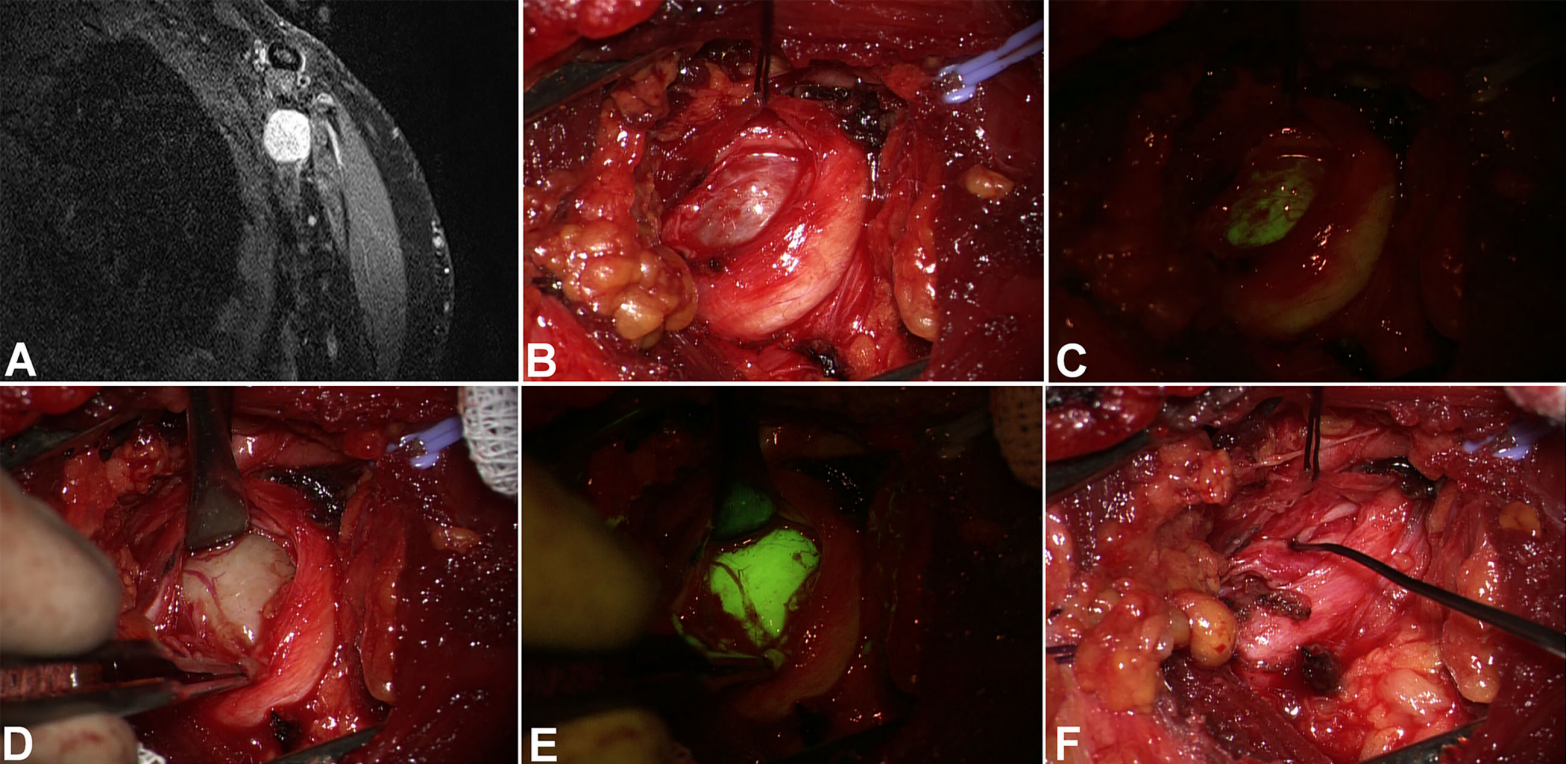
Left brachial plexus schwannoma (coronal MRI scan in **A**). After skin incision and subcutaneous tissues dissection, the tumor appeared surrounded by the nerve **(B)**. The first examination under YELLOW 560 filter **(C)** showed a slight and diffuse fluorescence both of the tumor and the nerve covering the tumor. However, after pseudocapsule incision **(D)**, the tumor showed an intense and homogeneous fluorescence **(E)**, whereas the nerve was only slightly fluorescent. Tumor removal was performed under intraoperative neurophysiological monitoring **(F)**.

**Figure 2:**
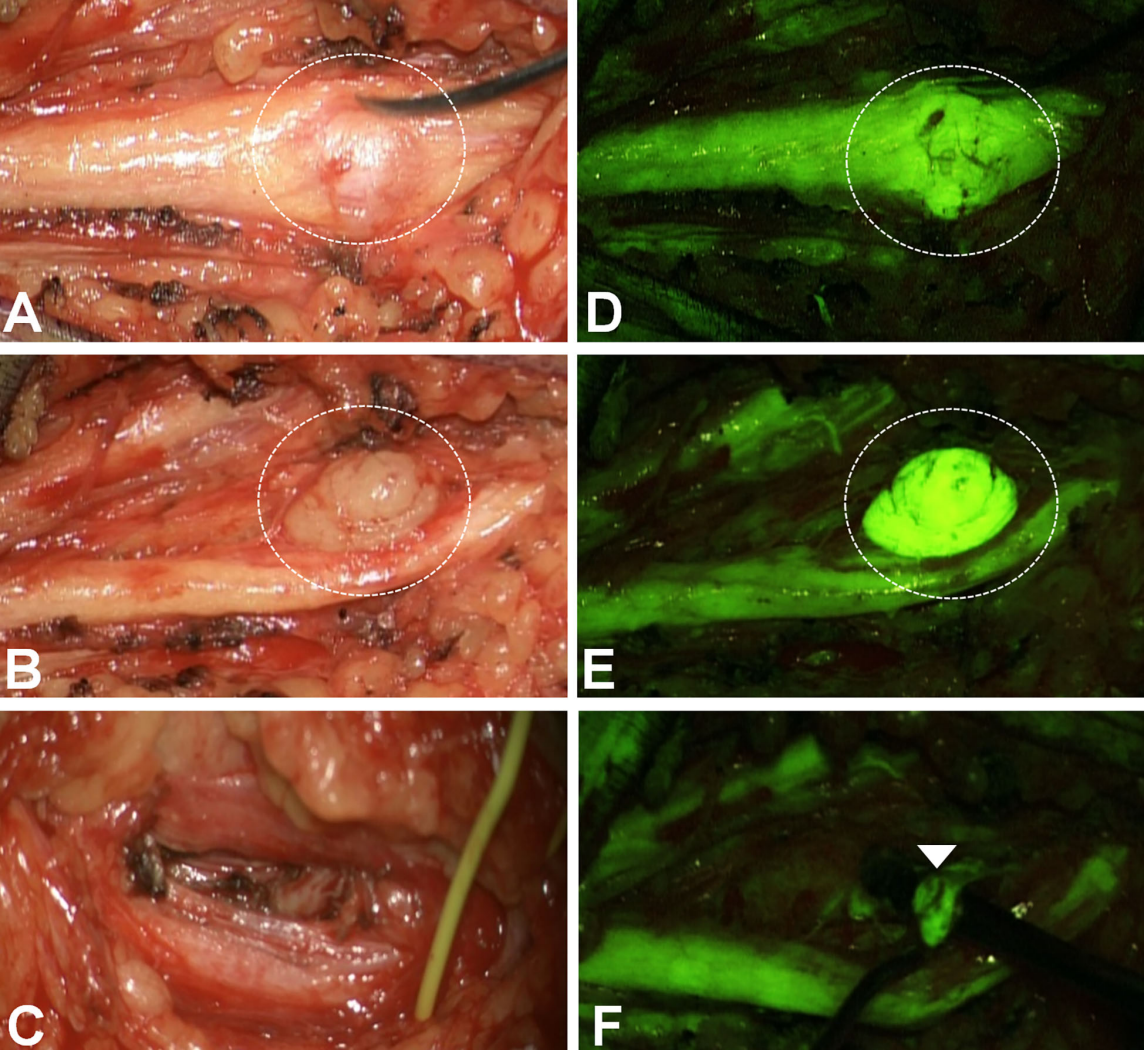
Remnants identification in a left superficial peroneal schwannoma (**A–C**: white light; **D–F**: YELLOW560). After the popliteal fascia opening, the sciatic-superficial popliteal nerve was identified and followed distally: one 1,5 cm schwannoma was visible (*dotted circle* in A). The first fluorescein examination **(D)** depicted a corresponding area of more slight fluorescence than the nerve. Therefore the capsule was opened **(B)**. However, the tumor appeared intensely fluorescent, more than the nerve **(E)**. After tumor removal **(C)**, the last examination under YELLOW560 evidenced a small remnant (*arrowhead*) non-clearly visible under white light, then removed to obtain a gross-total resection.

In 117 out of 142 patients (82.4%), we achieved a complete tumor removal (**Figure 1**), defined as radiological (at 3-months MRI) and histological radical excision without border infiltration at the histological specimen. We then analyzed these results concerning tumor histopathology. Similar complete resection rates in schwannomas (81/92) and neurofibromas (15/17) were achieved. In plexiform neurofibromas, the complete removal rate was 66.7% (8/12), while in MPSNT, 40% (2/5). In 6 cases of neurofibromas and in two schwannomas (**Figure 2**), the final YELLOW 560 visualization showed the presence of small remnants not visible under white-light illumination; this occurred more frequently in plexiform neurofibromas (4 of these 6 cases). The overall complication rate was 7.7% (11/142), considering both transient and permanent new neurological deficits. In 5 cases, a postoperative transient sensitive deficit was recorded, whereas all recovered completely at the three months follow-up. In four cases, postoperative worsening of a pre-existing motor deficit was present; after physio-kinesiotherapy, the patients partially recovered: in 2 cases, at the first follow-up visit, the neurological status was superimposable with the preoperative one, while in the other 2 cases a mild worsened motor deficit remained. A cerebrospinal fluid leak occurred in a sacral schwannoma with intradural extension, requiring revision surgery but without neurological impairment. Only one patient with a large cervical neurofibroma had a new motor deficit after surgery with left arm hyposthenia that persisted after physio-kinesiotherapy.

We finally compared these results retrospectively with a historical cohort of patients submitted to surgery between 2015 and 2018, matched for age, gender, and lesion characteristics. The “fluorescein” group (2018-2021) and the “standard of care (SOC)” group were comparable in terms of overall complete tumor removal and complication rates, the primary endpoints of our study. Looking at histological subgroups, we found a consistently higher rate of complete tumor removal in plexiform neurofibromas, 66% in the “fluorescent” group vs. 44% in the “SOC” group (p-value < 0.05, Fisher’s exact test, CI 95%) (**Table 2**).

**Table 2:** Clinical characteristics of the comparison historical cohort of PNST.

Histology	N°	Neurocutaneous syndromes	Location	Resection	Complications
**Schwannoma**	86 (60%)	5 Schwannomatosis 2 NF1 1 NF2	36 Inferior Limbs 23 Superior limbs 15 Intracanalar 12 Brachial Plexus	79/86 Total (92%) 7/86 Subtotal (8%)	1 permanent sensory deficit 1 permanent motor deficit 3 transient sensory deficits 2 transient motor deficits 2 dural tear
**Neurofibroma**	37 (26%)	27 NF1 1 Schwannomatosis	13 Superior limbs 7 Inferior limbs 5 Chest 4 Head 3 Brachial Plexus 3 Back 2 Intracanalar	36/37 Total (97%) 1/37 Subtotal (3%)	1 permanent sensory deficit
**Plexiform Neurofibroma**	9 (6%)	9 NF1	4 Inferior limbs 3 Superior limbs 1 Chest 1 Intracanalar	4/9 Total (44 %) 5/9 Subtotal (56 %)	1 permanent motor deficit
**Plexiform Schwannoma**	3 (2%)	none	2 Superior limbs 1 Inferior limbs	3 Total (100%)	1 transient sensory deficit
**Malignant Peripheral Nerve Sheath Tumor (MPSNT)**	2 (1.5%)	2 NF1	2 Brachial Plexus	2 Subtotal (100%)	no
**Fibrolipoma**	1 (0.7%)	none	Superior limbs	Total	no
**Branchial cyst**	1 (0.7%)	none	Brachial plexus	Total	no
**Lipoma**	1 (0.7%)	none	Neck	Total	no
**Nodular fascitis**	1 (0.7%)	none	Inferior limbs	Total	no
**Lymphoma**	1 (0.7%)	none	Neck	Total	no
**Total**	142	40 NF1 6 Schwannomatosis 1 NF2	49 Inferior limbs 42 Superior limbs 18 Brachial Plexus 18 Intracanalar 6 Chest 4 Head 3 Back 2 Neck	127/142 Total (89%) 15/142 Subtotal (11%)	12/142 (8.5%)

## 4 Discussion

In our surgical series, the utilization of SF has represented a helpful adjunct in most of the PNST, mainly in neurofibromas. Compared to the retrospective analysis of a historical cohort well-matched for demographic and clinical characteristics, the use of SF could add significant advantages to tumor visualization. In particular, SF constituted a valuable tool in increasing the extent of resection in plexiform neurofibromas. During the last years, SF has emerged as an intraoperative tracer able to improve brain-tumor visualization (16–18, 33, 34), due to its non-specific, vascular mechanism of action related to the accumulation in brain regions with blood-brain barrier (BBB) disruption, as it happens with MRI contrast enhancement (35). The availability of integrated and specific filters in the surgical microscope has contributed to the wide diffusion of fluorescein (36). Therefore, based on our extensive experience with CNS tumors, we empirically started this experience of fluorescent PNST using a low dosage of 1mg/kg of sodium fluorescein, i.v. injected during the patient intubation to achieve optimal discrimination between the tumor and normal surrounding nerves (25, 29). Moreover, another factor that led us to evaluate the role of SF in PNST was that other diffused intraoperative dye as 5-ALA has demonstrated, in few cases of spinal neurinomas, the lack of positive fluorescence pattern, in relation to its specific mechanism of action (37, 38).

**Figure 3:**
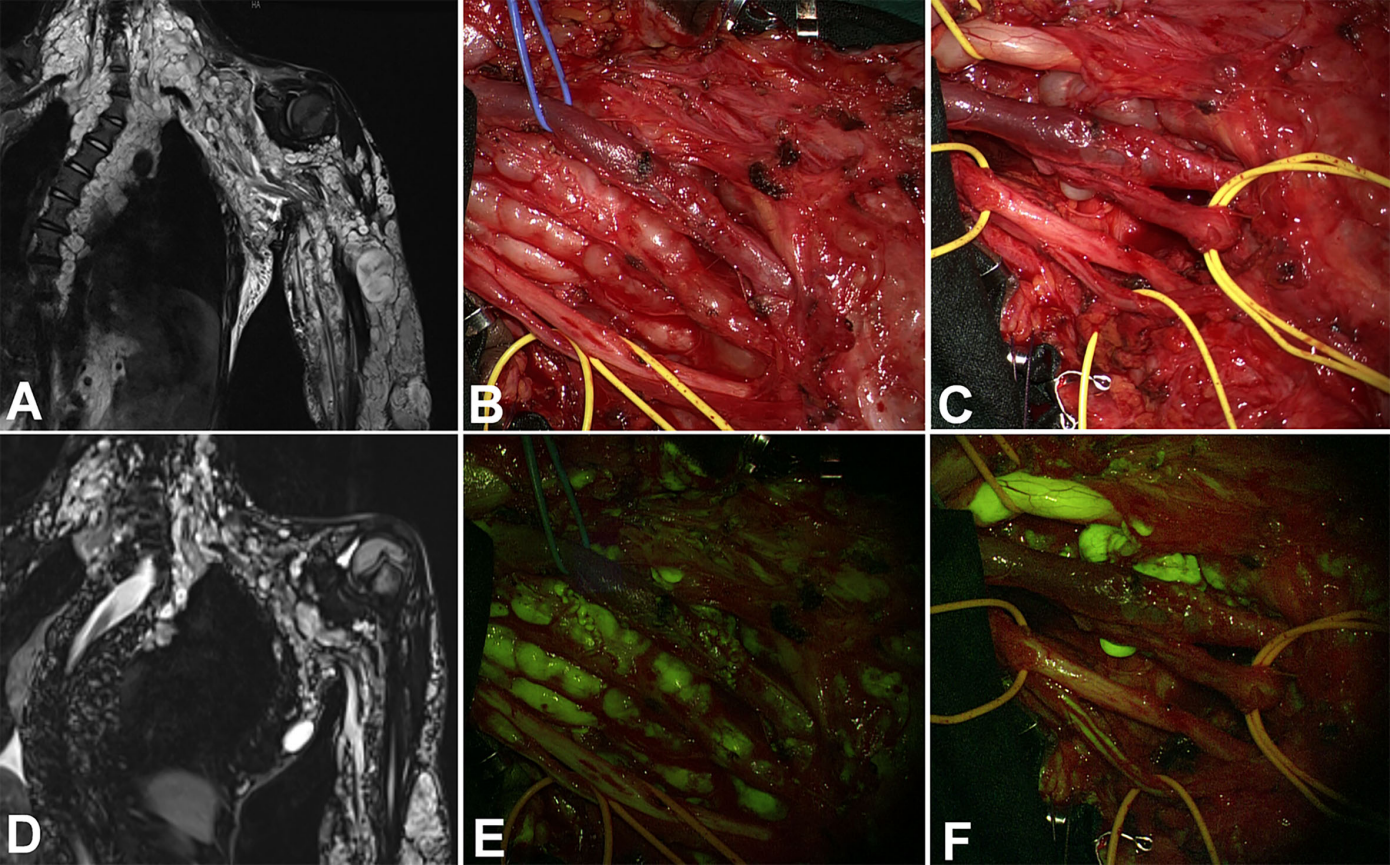
Coronal view in a huge plexiform neurofibroma of the whole upper left arm. Due to the rapid increase of a nodular lesion between radial and median nerves, the patients underwent partial surgery (coronal view in **(A)** – preoperative – and **D** – postoperative), with progressive exposure of the nerves by alternating the white light **(B, C)** and the YELLOW560 filter activation **(E, F)**. The tumor showed intense fluorescence if compared to the moderate fluorescein uptake of the nerve, whereas the neurofibroma partially substituted the radial nerve **(F)**.

In our preliminary analysis comprising only 20 lesions, we discussed that fluorescein plays a valuable role during surgical resection of schwannomas by highlighting the pathological tissue with a brighter fluorescent appearance than the surrounding nerves (25). Nevertheless, we noted that the most relevant advantage of SF was obtained mainly in neurofibromas helping identify diffuse tumor remnants (**Figure 3**). The German group led by Pedro found similar results: the authors examined 21 cases of PNST during surgery under SF at the low dosage of 0.5-1mg/kg (22, 23). An optimal distinction between tumor and surrounding nerves was observed in all 17 schwannomas scheduled for fluorescein-guided surgery, in 1 neurofibroma, and 1 MPNST. The authors also experienced the role of SF in fascicular biopsy of lesions involving the whole nerve segment (23, 39), finding usefulness in a case of B-cell lymphoma, whereas the fluorescein uptake in the MPNST was so widespread that the SF-contrast was judged useless (23). Moreover, they stressed the role of ImageJ, an open platform for digital video analysis, in objectifying SF enhancement, confirming the intraoperative impression of increased fluorescence of the PNST compared to healthy tissue. After these experiences, SF was established as a standard visualization tool in PNST surgery at the authors’ Institution (23). Some case reports have also demonstrated the application and usefulness of SF in other peripheral nerve diseases, such as intraneural ganglion cysts (24). In contrast to all the previously mentioned findings, Kalamarides et al. examined five schwannomas with a fluorescein dosage of 0.5mg/kg (40) without noticing any benefit deriving from filter activation in tumor visualization or pathological tissue discrimination from nerve fascicles, except for only 1 case. However, these different results could partially be explained by the dose and timing of SF administration: as for CNS tumors, the high discrimination assured by SF appears to depend on the injection time and dosage (30). Regarding other commercially available fluorophores, 5-aminolevulinic acid (5-ALA) has been employed in spinal tumors but without any positive fluorescence pattern in the case of schwannomas (37). To date, no other study has been reported regarding using 5-ALA in PNST surgery, including MPNST.

**Figure 4:**
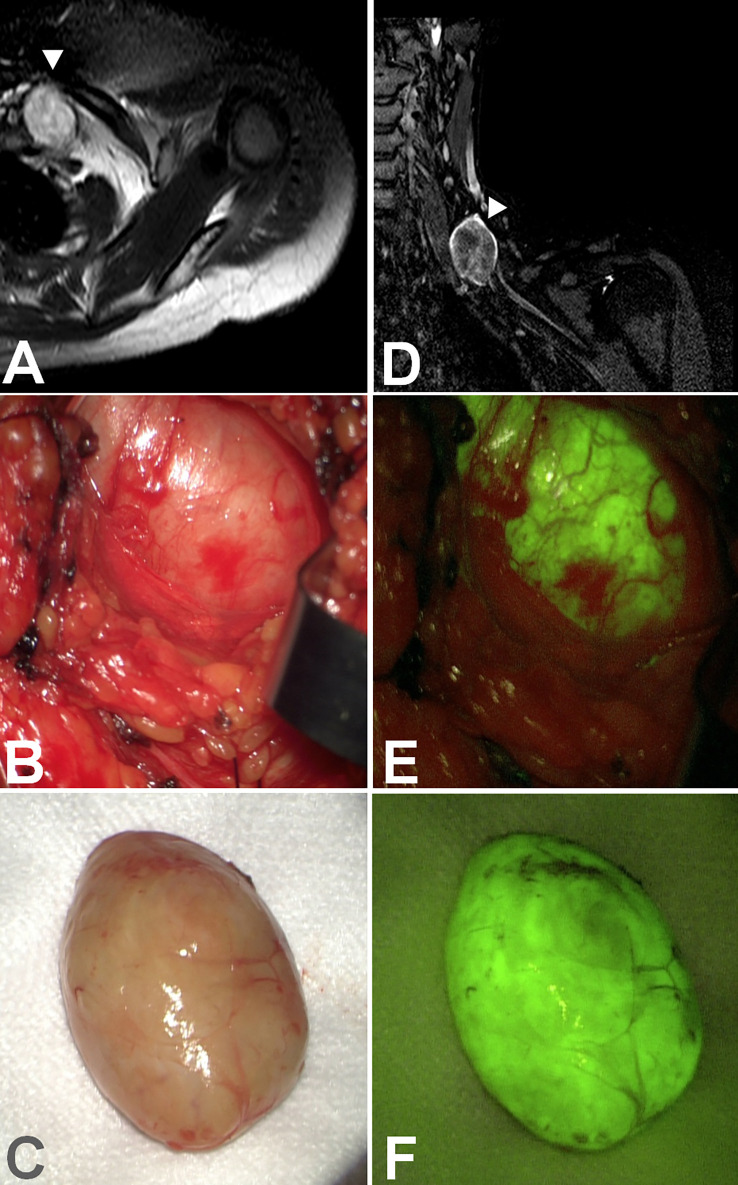
Axial **(A)** and coronal **(D)** view of a schwannoma of the suprascapular nerve. Under white light **(B)**, the tumor capsule was, in some parts, indistinguishable from the nerve, but the schwannoma showed intense fluorescein uptake **(E)** compared to the nerve lying lower. The tumor was removed en bloc **(C)** entirely, and intense and homogeneous fluorescence was still visible **(F)**.

A bright fluorescence was present in all schwannomas (**Figure 4**) and neurofibromas of the present series; on the contrary, the fluorescence pattern was significantly less evident for malignant PNST, as also reported by other groups (23). Perineurioma and hybrid nerve sheath tumors were instead characterized by a faint fluorescence enhancement, although the rarity of these histotypes cannot allow a strong generalization. Other tumors and lesions such as B-cell lymphomas, solitary fibrous tumors, or other malignant entities appeared highly fluorescent with an inhomogeneous pattern due to sporadic necrotic regions. Pedro and coworkers discussed about the role of a digitalized and video analysis of fluorescein enhancement: in our experience, comprising also CSN tumor oncology, we were used to evaluate a broad range of fluorescein enhancement due to several characteristics besides the specificity of the tumor, such as the timing of injection, previous radiation therapy or corticosteroid administration. Therefore, after an appropriate learning curve, we consider that an adequate subjective evaluation can overcome the software elaboration since the role of fluorescein magnification consists in allowing a discrimination between health and pathological fluorescent tissue, not based upon absolute uptake value how much rather on enhancement compared to the baseline of the surgical cavity.

**Figure 5:**
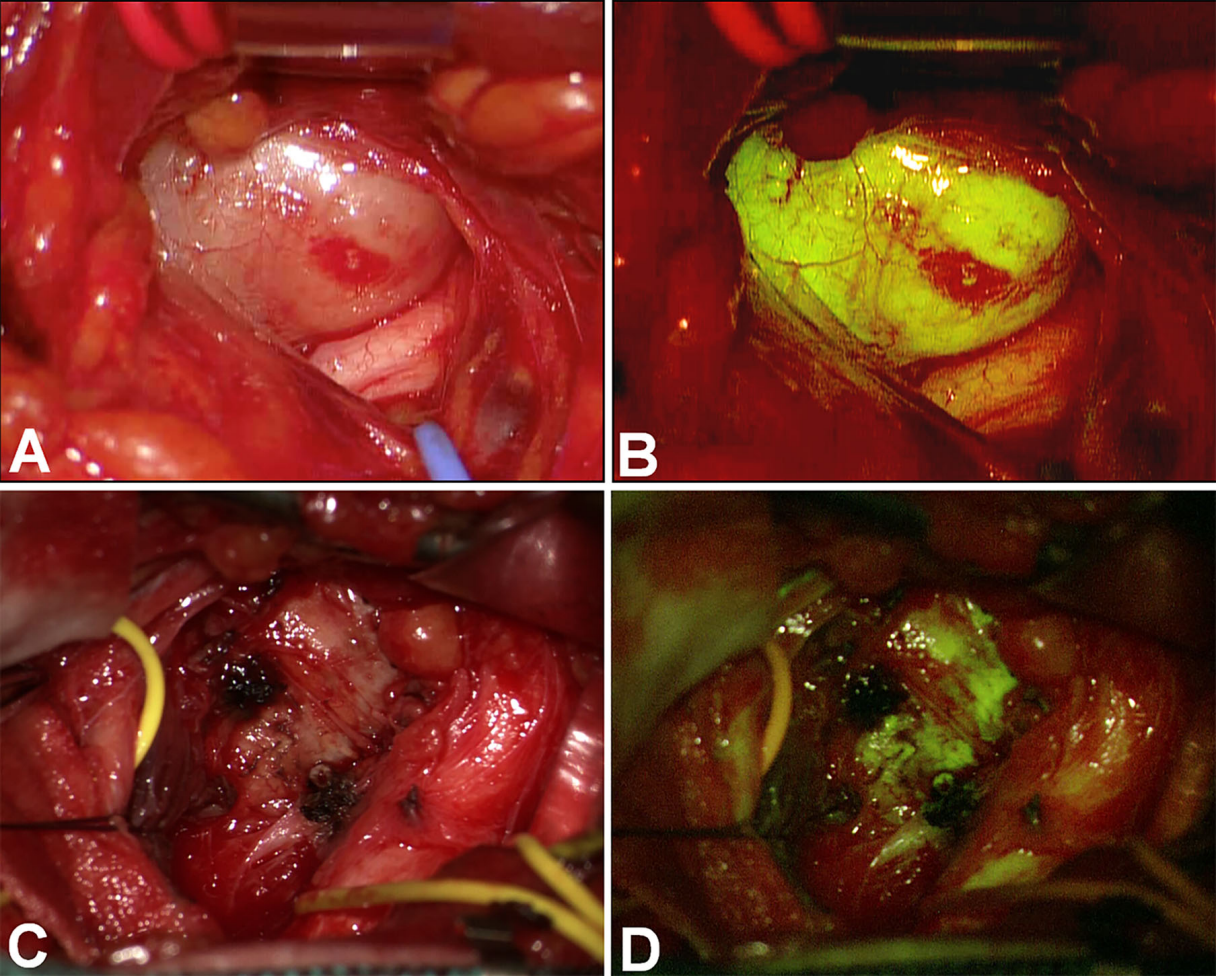
Comparison among the intense and homogeneous fluorescein uptake of a brachial plexus schwannoma **(A, B)** and a MPNST of the upper limb **(C)**, which showed inhomogeneous and slightly fluorescence **(D)**, compared to the surrounding nerve.

These uptake findings correlate to the characteristic’s enhancement of tumors at preoperative MR, and the concentration of SF depends on endoneurial extravasation. The key point is the disruption of the blood-nerve barrier (BNB), which shares some structural features with the BBB (26, 41). The interaction between blood and nerve sheath justifies our clinical findings, in particular the increase of fluorescent enhancement after pseudocapsule opening and during tumor resection: this evidence can be explained considering that tumor growth disrupts the tight junctions, a network of transmembrane and peripheral proteins, altering BNB structure and leading to fluorescein extravasation and accumulation inside neoplastic nodules (28). In particular, a stratified analysis of our results revealed that the contribution of fluorescein is more significant for schwannomas and neurofibromas: the former presented a bright fluorescence with intense delineation of tumor borders which made more straightforward surgery, especially for large lesions which need a piecemeal removal. In the case of neurofibromas, especially for the plexiform variant, the role of SF can be judged fundamental in increasing the resection rate, as demonstrated by the comparison to our historical institutional series. Differently from schwannomas, due to various entering and exiting fascicles in neurofibromas, SF facilitates progressive dissection and piecemeal resection, thus allowing further identification of small remnants not visible under white-light illumination. Most of surgery can be performed under YELLOW 560 visualization which allows to visualize the surgical field in similar natural color with an adequate brightness: therefore, we cannot be able to quantify the specific cases in which SF could have increased resection; this datum can be indirectly derived by means a comparison with the historical series. On the contrary, the use of fluorescein remains questionable for other rarer tumors, such as MPNST, showing a minimal or heterogeneous pattern of SF uptake (**Figure 5**) and widespread involvement of healthy and functional structures; further studies including specifically a single-tumor entity could determine the real potentiality of this methodic.

The complication rate is similar in the current series and the historical cohort. These findings can be explained by the role of IOM, employed for several years at our Institution, comprising both the control and the SF cohort. Intraoperative fascicular mapping and monitoring is still a surgical milestone; the use of SF represents, in our experience, an intraoperative adjunct without rejecting the standard principles of PNST microsurgical resection to preserve neurological function. We hypothesize the role of SF in selecting yellow nervous fibers silent at direct stimulation, probably due to tumor infiltration, to potentially increase surgical radicality. We did not report fluorescein-related side effects or adverse reactions. The only visible manifestation of fluorescein administration is the onset of a transient and harmless yellowish urinary stain that rapidly disappears after 24 hours. Despite the very high safety profile of SF, as also reported for ophthalmological applications that usually employ a dose of 500 mg (20, 42), the lack of side effects may be related to the low dosage used in this study, thanks to the dedicated filter into the microscope, that allowed more accurate identification of fluorescent tissue. Nonetheless, the main limitation of the presented study is represented by the heterogeneous histology of PNST included. In addition, the lack of a significant long-term follow-up prevents the elucidation of a possible correlation between the use of SF, the extent of resection, and survival. Another selection bias is the association of both sporadic and syndromic tumors. Future prospective studies could better address these limitations by stratifying the cohorts according to the major predicting factors of PNST removal, including tumor histology.

## 5 Conclusion

Fluorescein-guided surgery seems to be a safe and effective technique that can be used during the surgical resection of PNST to identify better and distinguish the most frequent subtypes of PNST from intact functional nerves. Prospective studies with long-term follow-up and designed for specific histologies could provide significant insights into the effects of fluorescein application on PNST patients’ outcomes.

## Data availability statement

Publicly available datasets were analyzed in this study. This data can be found here: https://zenodo.org/.

## Ethics statement

The studies involving human participants were reviewed and approved by Ethics Committee Fondazione IRCCS Istituto Neurologico Carlo Besta. The patients/participants provided their written informed consent to participate in this study.

## Author contributions

VN, NI, JF, NC, and IV: study concept and design. VN, NI, JF, IT, and IV: critical revision of the manuscript for intellectual content. All authors: acquisition of data, data analysis, and interpretation. VN and IV: study supervision. All authors contributed to the article and approved the submitted version.
